# Influence of surface nanotopography and wettability on early phases of peri-implant soft tissue healing: an in-vivo study in dogs

**DOI:** 10.1186/s12903-023-03347-7

**Published:** 2023-09-08

**Authors:** Caiyun Wang, Xin Wang, Ran Lu, Xu Cao, Dingxiang Yuan, Su Chen

**Affiliations:** Laboratory of Biomaterials and Biomechanics, Beijing Key Laboratory of Tooth Regeneration and Function Reconstruction, Beijing Stomatological Hospital, Capital Medical University, Beijing, 100050 China

**Keywords:** Peri-implant soft tissue, Collagen fibers, Nanotubes, Superhydrophilicity, Titanium alloy, Abutment

## Abstract

**Background:**

It is well established that nanotopography and wettability of implant surfaces contribute to osseointegration and long-term implant success. However, the effects of a hydrogenated surface with nanotubular and superhydrophilic properties on peri-implant soft tissue remain unclear. This study was designed to study the impact of a modified abutment surface on early soft tissue integration compared with a machined surface.

**Methods:**

Thirty-six implants were placed at the bone level in the bilateral mandible of six beagles, followed by healing abutments belonging to the standard machined Ti-6Al-4V alloy abutments (TC4-M), anodized abutments with nanotubes (TC4-Nano), and hydrogenated abutments (TC4-H/Nano) groups, which were randomly screwed to the implants. After two and four weeks of wound healing, the animals were euthanized for histological evaluation.

**Results:**

A superhydrophilic nanotubular surface developed on the hydrogenated abutment. Histological and histometric analyses revealed similar peri-implant soft tissue healing and dimensions for the three types of abutments at two and four weeks. Connective tissue (CT) length was longer around TC4-H/Nano abutments compared with standard abutments; however, the differences were not statistically significant. Moreover, collagen fibers in the TC4-H/Nano group extended and were attached perpendicularly to the superhydrophilic surface.

**Conclusions:**

Our results revealed that the soft tissue interface adjacent to the hydrogenated abutment is comparable to that of the machined abutment. A tendency of increased CT length and perpendicular collagen fibers was observed around the modified abutment. This study suggests that nanotubular/superhydrophilic surfaces could be a promising modification to enhance soft tissue sealing. However, comprehensive studies should be conducted to evaluate the peri-implant soft tissue around the modified abutment immunohistochemically, histopathologically, and clinically.

## Background

Titanium and its alloys are widely applied to dental implants due to their high corrosion resistance, mechanical properties, and excellent biocompatibility. The Ti-6Al-4V alloy (TC4) is of particular interest in clinical applications, considering its better density, elastic modulus, and mechanical strength than titanium [1, 2]. While Ti-6Al-4V implants in the market have achieved high clinical success, especially regarding osseointegration, soft tissue integration has been more challenging. The implant-soft tissue interface is fragile due to weak connective tissue (CT) attachment and epidermal downgrowth, leading to bacterial infiltration, tissue inflammation, and ultimately, marginal bone loss (MBL) [3, 4]. Therefore, sound soft tissue and marginal bone are beneficial for the long-term clinical stability of dental implants.

Previous investigations attempted different strategies to improve the soft tissue integration. López-López et al. [5] demonstrated that anatomic healing abutments are conducive to the maintenance of peri-implant soft tissue and marginal bone level in comparison to concave–straight abutments. Although Sinjari et al. [6] did not find less MBL around a scalloped implant shoulder, it must be noted that the festooned implant neck with support for the soft tissues could be important for aesthetics.

In addition to the design of transmucosal portion, different surface modifications have been proposed to achieve better barrier function of peri-implant soft tissue [7]. One principle is based on nano-modification of implant abutments to promote soft tissue integration. Electrochemical anodization has been applied to imparting nanotopography, including nanotubes, nanograss, nanopores, and nanotemplates to Ti implants [8]. Gulati et al. [9] proved that aligned nanopores on the micro-grooved titanium surface selectively enhanced the activity of osteoblasts and fibroblasts. Similarly, other in vitro studies have suggested that nanostructured surfaces enhance the adhesion and initial growth of epithelial cells and fibroblasts [10–12]. Nanostructured morphology, mechanical strength, electroactivity, and adsorption capacity of nanotubes might be useful for the treatments in dentistry [13, 14]. More importantly, hydrophilicity plays an important role in wound healing, leading to faster protein adsorption, cell adhesion, and improved tissue integration [15–17]. Matter et al. [18] demonstrated that a one-step synthesis of nanoarchitected and superhydrophilic coatings promoted fibroblasts adhesion and exhibits antimicrobial properties.

Although several studies have concentrated on the interactions between different kinds of cells and biomaterial surfaces, few studies have established the effects of surface nanotopography and wettability on peri-implant mucosa healing in vivo. A previous study demonstrated that a hydrophilic surface with nanocrystalline diamond decreased inflammatory responses and stimulated cell proliferation during the initial healing phase [19]. Recently, a clinical study showed a thicker CT portion at abutments with plasma treatment, which could be attributed to bioactivation and increased wettability of the abutment surface through plasma [20]. Despite the different efforts, the implant-soft tissue interface could not rival the natural periodontal tissue.

As previously shown, hydrogenated nanotubes with superhydrophilicity have been verified to promote adhesion, migration, and extracellular matrix (ECM) synthesis of gingival fibroblasts in vitro [21]. However, the biological effect of this modified surface on peri-implant soft tissue in vivo remains unclear. In addition, limited information about Ti-6Al-4V alloy modified with nanostructure and superhydrophilicity applied as an implant abutment in vivo has been provided.

Thus, this study aimed to assess soft tissue healing around Ti-6Al-4V alloy abutments with nanotubes and superhydrophilic surfaces regarding histometric outcomes and collagen fiber orientation during the early stages of wound healing in a canine model.

## Methods

### Abutment preparation

**Fig. 1:**
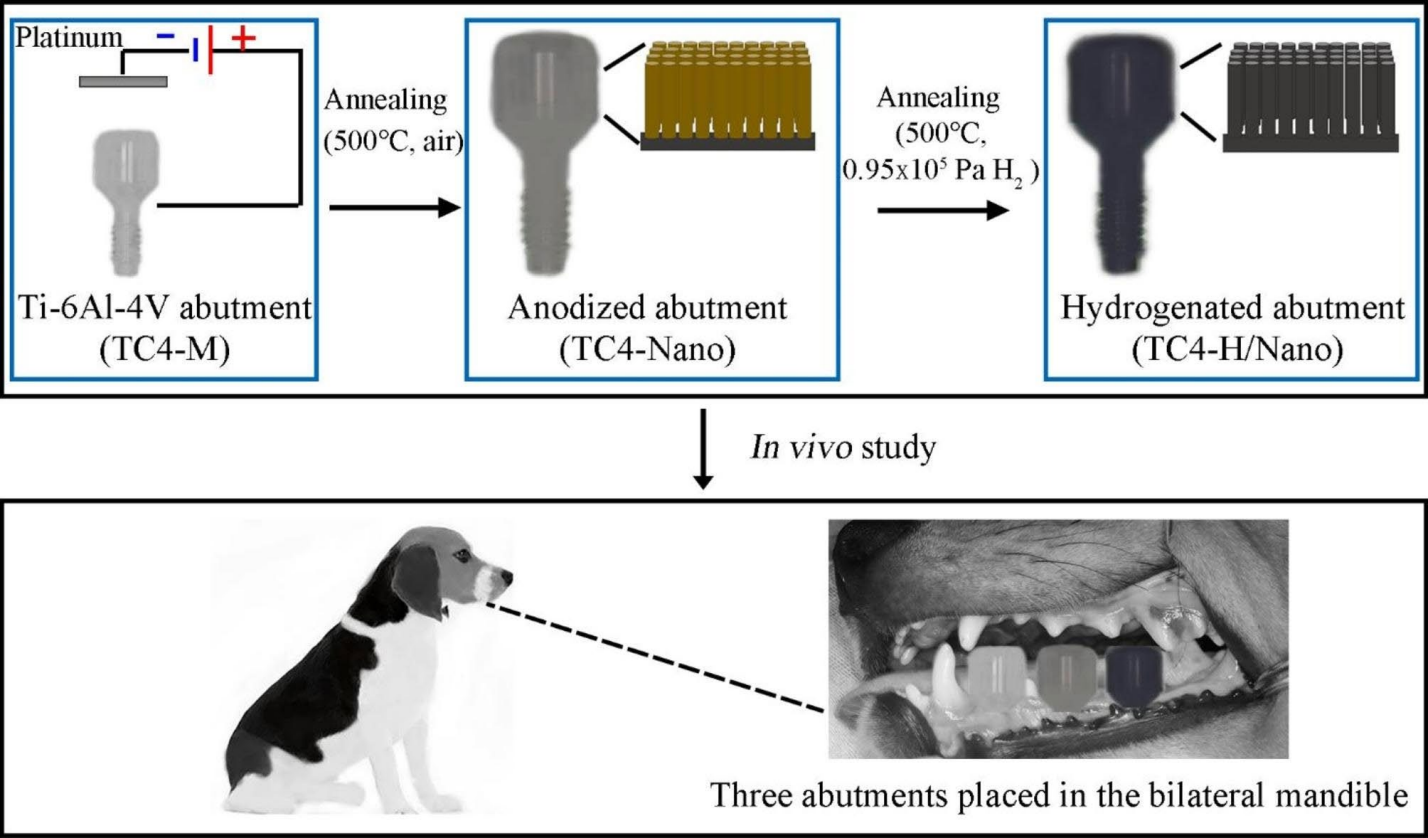
The diagram summarising the design of this study.

The diagram summarising the design of this study is displayed in Fig. 1.

Healing abutments (Ti-6Al-4V, TC4) with a 3.5 mm platform and 3 mm cuff height (Zimmer Biomet Dental, HC333) were used. Standard abutments with machined surfaces were used as the control group (TC4-M). Anodized abutments with nanotubes (TC4-Nano) and hydrogenated abutments (TC4-H/Nano) were produced as previously described [15]. Briefly, standard abutments were oxidation anodized in ethylene glycol at 50 V for 15 min and annealed at 500 °C for 2 h in air. After ultrasonication, the TC4-Nano was obtained. Subsequently, the anodized specimens were annealed in hydrogen (0.95 × 10^5^ Pa) at 500 °C for another 4 h to prepare hydrogenated abutments (TC4-H/Nano).

Twelve samples of each group (TC4-M, TC4-Nano, and TC4-H/Nano) were used for the subsequent characteristic assessments, and another twelve samples of each group were sterilised by autoclaving and used for the in vivo experiments.

### Characterization of the specimens

For surface morphology observation, field-emission scanning electron microscopy (FE-SEM; S4800, Hitachi, Ltd., Tokyo, Japan) was performed on the different groups of abutments. An accelerating voltage of 15 kV was chosen, and the SEM images were recorded from the surfaces at normal incidence [9] under high vacuum by a secondary electron detector. Elemental analysis of the abutment surface was performed using SEM-energy dispersive X-ray spectroscopy (SEM-EDS). Chemical elements were detected by X-ray photoelectron spectroscopy (XPS; ESCALAB 250Xi, Thermo Fisher Scientific, MA, USA) [22]. The surface roughness was analyzed using atomic force microscopy (AFM; Nanoscope V, Veeco Plainview, NY, USA). Briefly, three randomized areas (5 μm × 5 μm) were characterized on each sample (three samples per group) to get the average surface roughness (Ra), which was calculated by software NanoScope Analysis 1.8 (Bruker, Karlsruhe, Germany) [23]. An optical contact angle measuring device (Model OCA15pro, Dataphysics Co., Ltd., Germany) was employed for water contact angle measurements [24]. In short, 2 µL of distilled water was dropped on the surface, and a picture was captured using a high-resolution camera. Then the contact angle was analysed by ellipse fitting method. Each sample was tested three times, and three samples were detected in each group.

### Study animals

Six lab-bred male beagles (Fangyuanyuan Co., Ltd, Beijing, China) approximately 1 year of age were used for this study. All beagles were randomly divided into two groups. Three beagles in Group A were observed for 2 weeks after placement of the healing abutments, while three beagles in Group B were allowed to heal for 4 weeks. It has been reported that epithelial healing around teeth and dental implants is achieved in 1–2 weeks [25], and the integrity of soft tissue wound and the organization of collagen fibers are nearly accomplished in 4–6 weeks [26, 27]. Thus, two time points (2 and 4 weeks) were selected in this study to investigate early stages of soft tissue healing around Ti-6Al-4V alloy abutments.

This study was conducted in accordance with a protocol approved by the ‘Animal Ethical and Welfare Committee’ of Beijing Stomatological Hospital, Capital Medical University. All procedures were conducted in accordance with the Guiding Principles for Research Involving Animals. The animals were individually housed at the Laboratory Animal Center of Beijing Stomatological Hospital, Capital Medical University (Beijing, China). All beagles were acclimatized for 2 weeks prior to the surgeries. No animals were lost during the study. This manuscript was prepared in accordance with the ARRIVE (animal research: reporting of in vivo experiments) guidelines for animal research reports.

### Dental implants and abutments

**Fig. 2:**
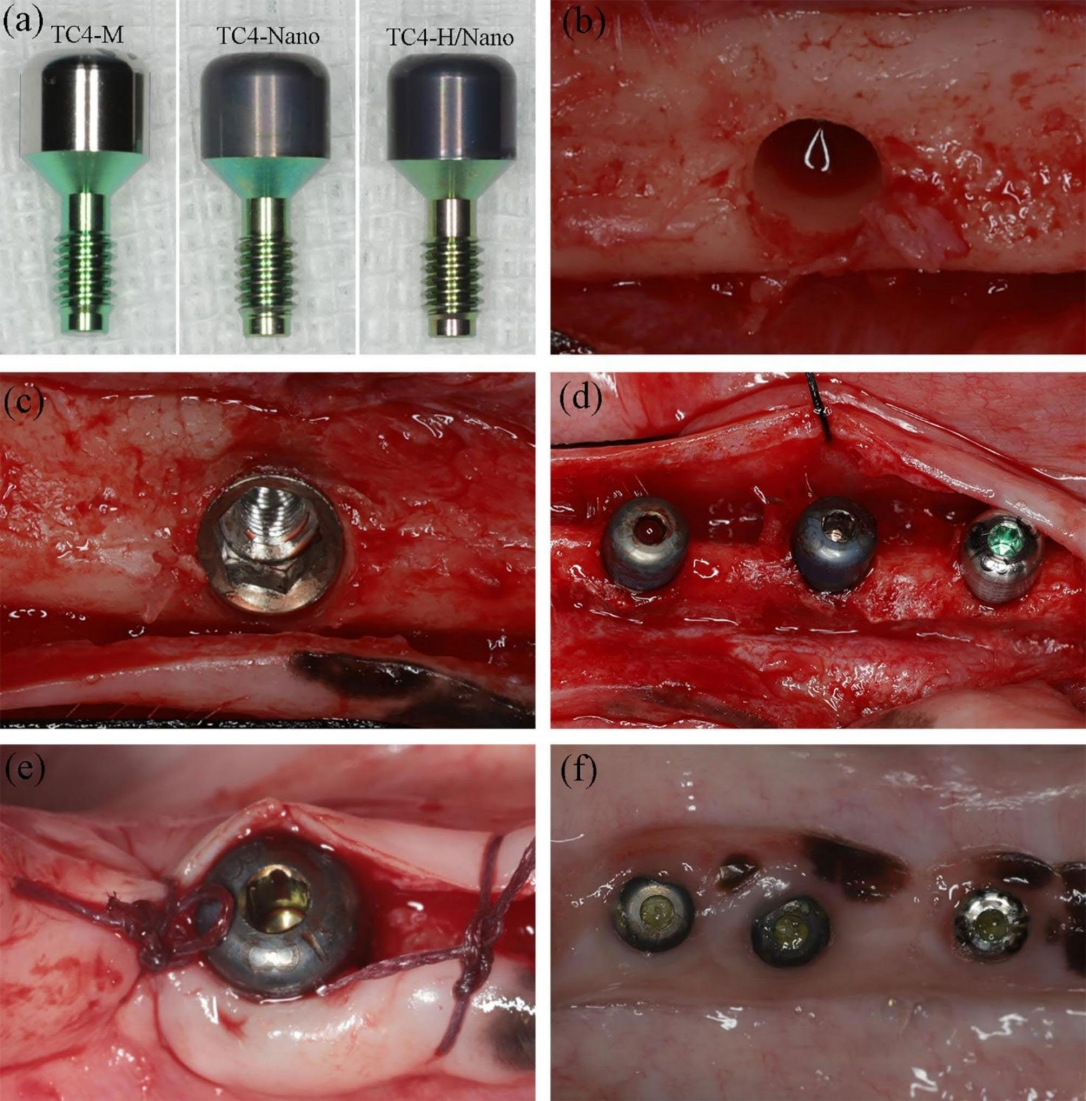
Clinical pictures illustrating the experimental surgery. **(a)** Experimental groups: standard machined abutment (TC4-M), anode oxidized abutment (TC4-Nano), and hydrogenated abutment (TC4-H/Nano). **(b)** At 3 months after tooth extraction, the osteotomy site was prepared to the desired diameter for implant placement. **(c)** TSV implants were placed bilaterally in the lower jaws (6 implants per animal). **(d)** Healing abutments were immediately connected (from left to right: TC4-Nano, TC4-H/Nano, TC4-M). **(e)** The flaps were mobilized with interrupted sutures. **(f)** The three abutments and surrounding mucosa at 4 weeks of healing. (from left to right: TC4-Nano, TC4-H/Nano, TC4-M).

Zimmer Tapered Screw-Vent (TSV) implants with a microtextured surface of 3.7 mm diameter and 8 mm length (Zimmer Biomet Dental, TSVB8) were used for all groups. Six TSV implants, three in each hemimandible, were placed per beagle. After that, three healing abutments from each group (TC4-M, TC4-Nano, and TC4-H/Nano, as shown in Fig. 2a) were installed in each hemimandible based on a computer-generated randomization chart, negating the possibility of a biased abutment position in the hemimandible. There were six experiments in each group at each time point. Based on the literature and previous experience, we estimated that a significance level of 5% would be achieved with n = 6.

### Surgeries

#### Extraction

The food was restrained 12 h before the surgeries. All surgical procedures were conducted by the same surgeon. All surgeries were conducted under general anesthesia with a mixture of Sumianxin II (0.08–0.1 mL/kg, Jilin Huamu Animal Care Products, Ltd, Jilin, China) and 3% pentobarbital sodium (0.5 mL/kg, Beijing Daniel Spulber Biotech, Beijing, China) via intramuscular administration. Subsequently, 1.7 mL of 4% articaine with 1:100,000 adrenaline (Septanest; Septodont, Cholet, France) was used for local anesthesia.

Extractions of the bilateral mandibular premolars were performed as described in a previous article [28]. Briefly, the premolars were sectioned in buccolingual directions, followed by nontraumatic extractions with root elevators and forceps. The sockets were then sutured with collagen sponges. Following the surgical procedures, beagles received benzylpenicillin sodium (40,000 IU/kg, Shandong Shengwang pharmaceutical Co., LTD, Jining, China) via intramuscular administration for three days and a soft diet was used for 1 week. The extraction sockets were allowed to heal for three months.

#### Implant and healing abutment placement

After three months of healing, the implants were placed following the manufacturers’ recommendations.The procedure used for implant placement has been described in detail in another study [29]. Briefly, crestal incisions were made, followed by the elevation of the mucoperiosteal flaps. After flattening the edentulous ridge, spaces between implants were measured, and the osteotomy sites were prepared under chilled normal saline solution (Fig. 2b). Three implants were placed in each hemimandible, with the buccolingual implant shoulder at the marginal bone crest level (Fig. 2c). Next, three healing abutments from each group were installed based on a computer-generated randomization chart (Fig. 2d). The flaps were approximated using interrupted sutures (Fig. 2e). The implants and abutments remained in vivo for two or four weeks prior to animal sacrifice. The postoperative protocol used for implant placement was the same as for tooth extraction.

No implants were lost during the experiment period, and no signs of peri-implant inflammation were observed. Figure 2f shows the three abutments and surrounding soft tissue at four weeks of healing.

### Histological preparation

After two or four weeks of healing, the animals were sacrificed using an overdose of pentobarbital sodium (100 mg/kg). ​Histological procedures were carried out based on previously published articles [30, 31]. In short, mandibular segments were dissected into blocks containing implants, abutments, and the ambient hard and soft tissues. The blocks were immersed in a solution of 10% formalin for 24 h and then washed with current water for 24 h. After dehydration in graded ethanol, the blocks were embedded in methylmethacrylate and sectioned into buccolingual slices, aiming for the center of the implant and abutment along the long axis. The slices were ground and polished to a final thickness of 30–40 μm. The obtained sections were stained with Van Gieson’s stain and analyzed using a light microscope (BX51; Olympus, Tokyo, Japan).

### Histologic and histometric analyses

**Fig. 3:**
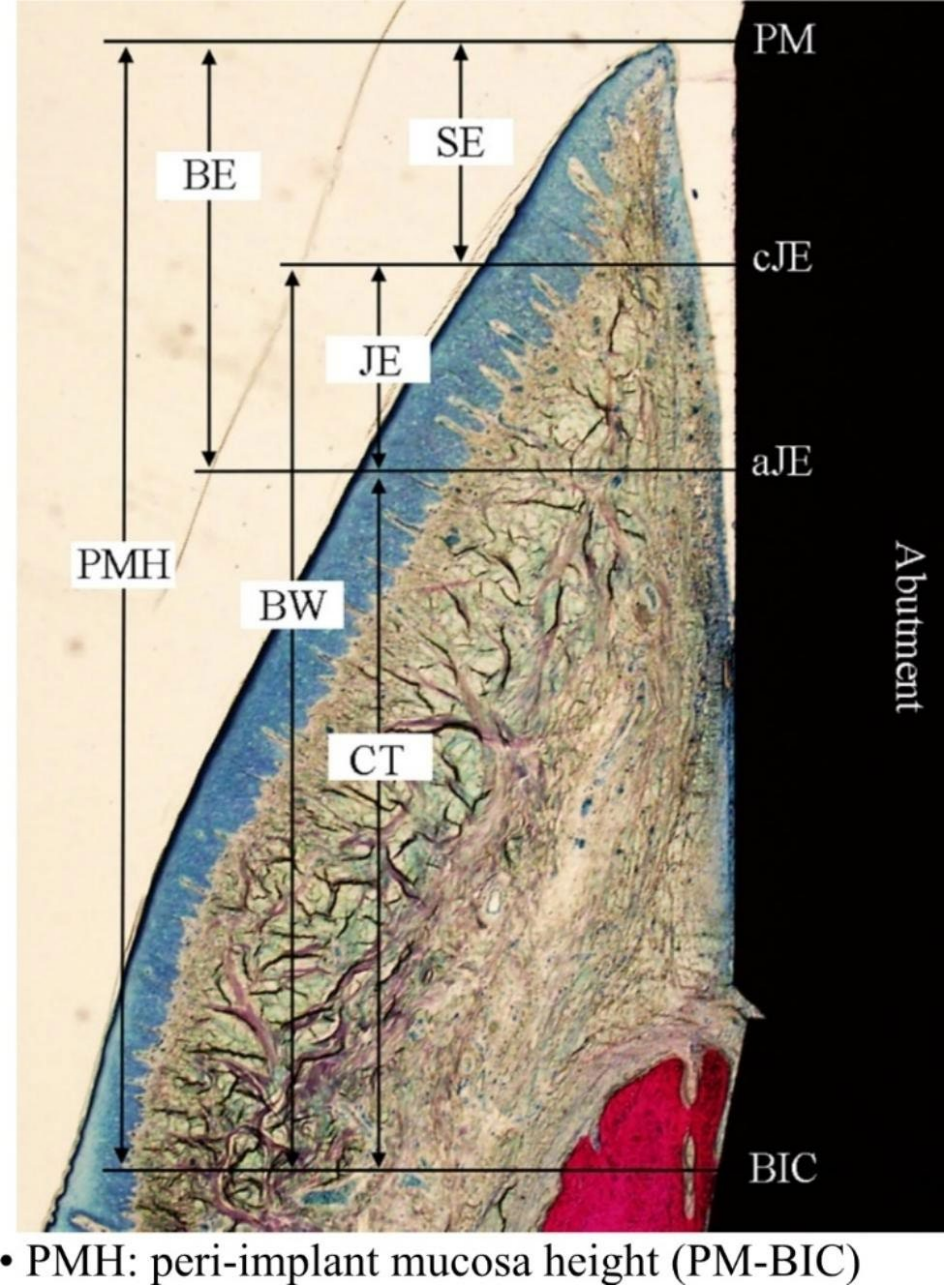
Landmarks for histometric evaluation. PM, peri-implant mucosa margin; cJE, most coronal point of the junctional epithelium; aJE, apical extension of the junctional epithelium; BIC, most coronal portion of the bone-implant contact. Linear measurements between landmarks: SE, length of sulcular epithelium (PM-cJE); JE, length of junctional epithelium (cJE-aJE); BE, barrier epithelium consisted of the dimension of SE and JE (PM-aJE); CT, length of connective tissue (aJE-BIC); BW, biological width consisted of the dimension of JE and CT (cJE-BIC); PMH, peri-implant mucosa height (PM-BIC).

Histologic and histometric outcomes were observed by the same experienced investigator using ImageJ software. The following landmarks were designated in the buccolingual sections (Fig. 3) according to a method published previously [32]:

- PM: peri-implant mucosa margin.
- cJE: most coronal point of the junctional epithelium.
- aJE: apical extension of the junctional epithelium.
- BIC: most coronal portion of the bone-implant contact.

Linear measurements between landmarks were performed by drawing a vertical line along the long axis of the abutment. The measurements were performed as follows:

- SE: length of sulcular epithelium (PM-cJE).
- JE: length of junctional epithelium (cJE-aJE).
- BE: barrier epithelium consisted of the dimension of SE and JE (PM-aJE).
- CT: length of connective tissue (aJE-BIC).
- BW: biological width consisted of the dimension of JE and CT (cJE-BIC).

### Collagen fiber orientation

To detect collagen fiber distribution and orientation in the CT surrounding the abutments, the sections were analyzed using second harmonic generation (SHG) microscopy (Olympus, Japan). The excitation wavelength was 890 nm, and images of the SHG signal were collected using a 445 nm detection barrier filter to observe the collagen fibers [33].

### Statistical analysis

The variables are expressed as mean values and standard deviations (SDs). IBM SPSS Statistics 19.0 software (International Business Machines Corporation, NY, USA) was used for statistical analysis. The Kolmogorow-Smirnow test was applied to analyze the normal distribution of the data rows. Multiple comparisons were performed using one-way analysis of variance (ANOVA) When the homogeneity variance of the data was not assumed, Kruskal-Wallis test was used [34]. *P* < 0.05 was considered statistically significant.

## Results

### Characterization of the specimens

**Fig. 4:**
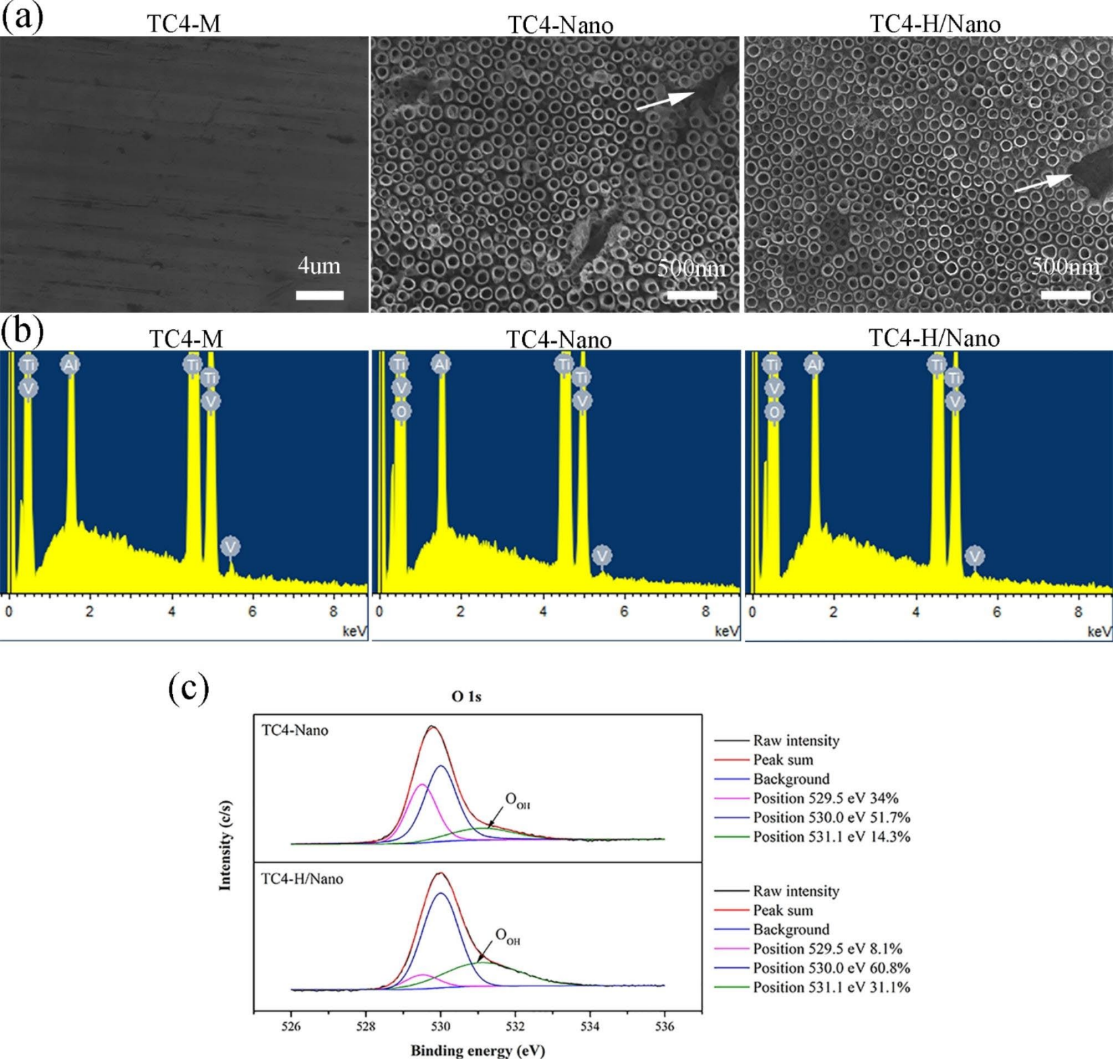
Characterizations of the specimens. **(a)** Scanning electron microscopy (SEM) images of the TC4-M abutment surface at low magnification (×3000); the TC4-Nano abutment surface at high magnification (×30,000); and the TC4-H/Nano abutment surface at high magnification (×30,000). White arrows: shorter nanotubes formed in the β-phase. **(b)** Energy dispersive X-ray spectroscopy (EDS) spectra of the TC4-M, TC4-Nano and TC4-H/Nano. **(c)** High-resolution O 1s peaks of X-ray photoelectron spectroscopy (XPS).

The surface morphologies of all specimens were characterized by SEM. As shown in Fig. 4a, the machined abutment (TC4-M) surface was relatively smooth with regular mechanical polishing scratches, whereas TC4-Nano and TC4-H/Nano showed non-uniformly arranged nanotube arrays because of the existence of α and β phases in the TC4 alloy, with a diameter of approximately 100 nm. Shorter nanotubes were formed in the β-phase (Fig. 4a, white arrows). Significantly, the original nanotubular geometry was unaltered after the thermal hydrogenation treatment.

The EDS spectra are shown in Fig. 4b, and the quantitative results are presented in Table 1. The TC4-M abutment consisted of Ti, Al, and V. In addition, O was observed in the TC4-Nano (62.88%) and TC4-H/Nano (60.4%). The TC4-H/Nano group showed a percentage decrease in O compared to TC4-Nano due to thermal hydrogenation.

**Table 1:** Elemental surface composition (at%) of differently modified TC4-M, TC4-Nano and TC4-H/Nano abutment surfaces (mean ± SD).

Material	% Ti	% Al	% V	% O
TC4-M	86.10	10.61	3.29	-
TC4-Nano	32.22	3.64	1.26	62.88
TC4-H/Nano	34.60	3.81	1.19	60.40

To further characterize the chemical valence environment, the O 1s XPS spectra of TC4-Nano and TC4-H/Nano were compared (Fig. 4c). The O 1s peaks can be decomposed into three peaks; the peaks at 529.5 and 530.1 eV are ascribed to Ti-O species on the surface, and the broader peak at 531.1 eV corresponds to Ti-OH [35, 36]. The percentage of O_OH_ atoms increased from 14.1 to 31.1% after hydrogenation.

**Fig. 5:**
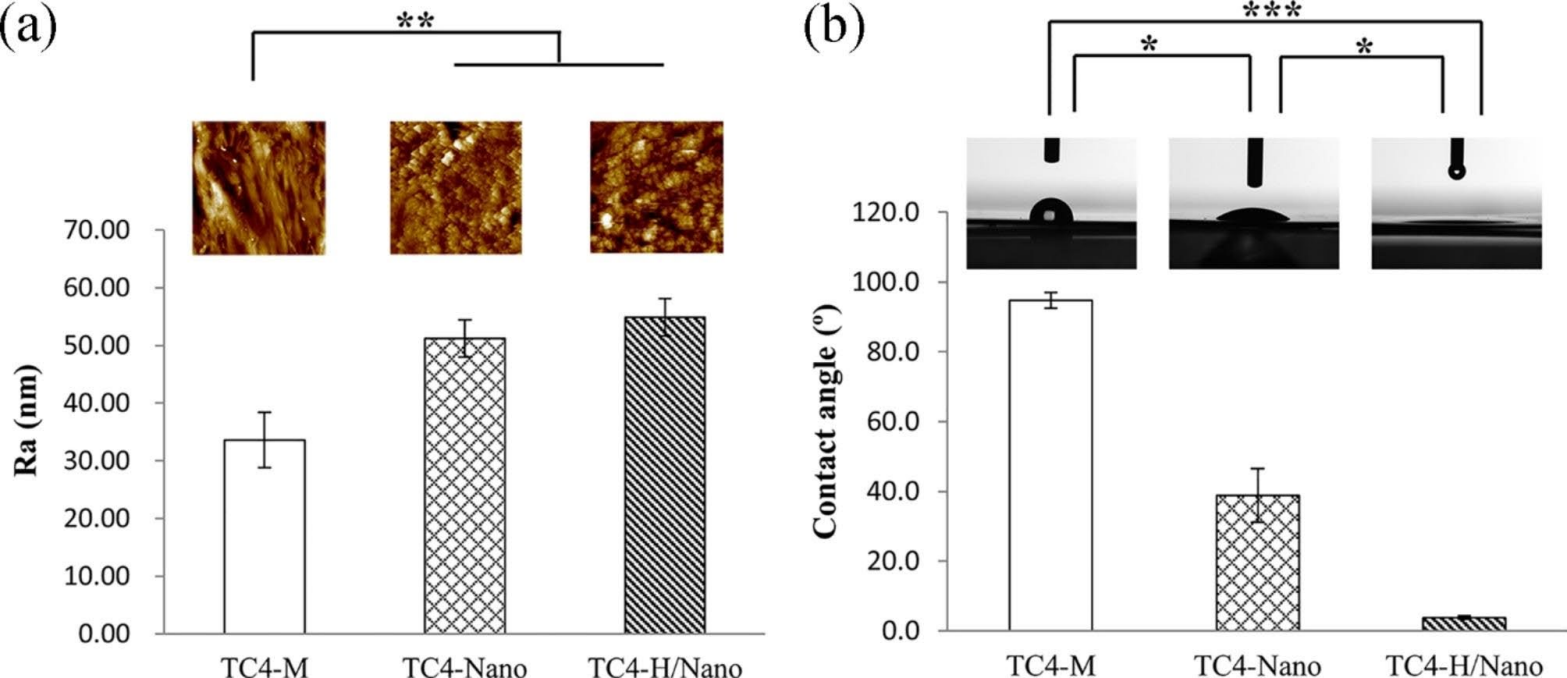
**(a)** Surface roughness of the specimens. **(b)** The water contact angle of the specimens

As shown in Fig. 5a, the roughness of nanotopography significantly increased compared with the machined abutment (*p* < 0.01), whereas the roughness of TC4-H/Nano and TC4-Nano was not significantly different (*p* > 0.05). The average contact angles of all the groups are displayed in Fig. 5b. The TC4-M group featured a hydrophobic surface (94.7°), whereas the contact angle of TC4-Nano was reduced to 38.8°. Notably, TC4-H/Nano displayed a superhydrophilic surface with a contact angle of 3.8°.

### Histology

**Fig. 6:**
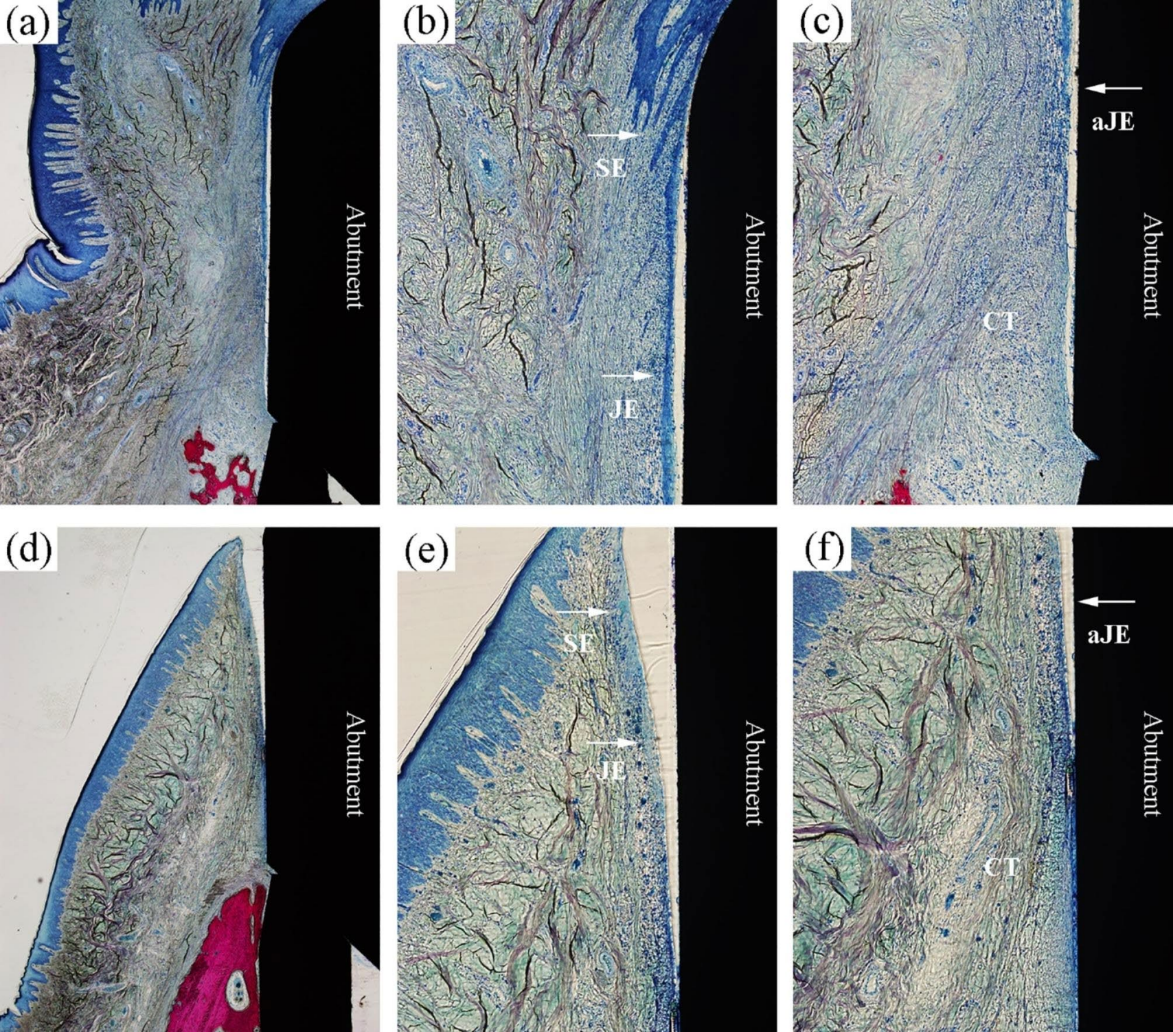
Histologic images of peri-implant soft tissue in TC4-H/Nano group representing **(a, b, c)** 2 weeks and **(d, e, f)** 4 weeks of healing. **(a, d)** original magnification ×40. **(b, c)** Details of **(a)**, original magnification ×100. **(e, f)** Details of **(d)**, original magnification ×100. SE, sulcular epithelium; JE: junctional epithelium; aJE: apical termination of junctional epithelium.

Histological examination of the peri-implant mucosa adjacent to different abutments was performed using an optical microscope. Similar observations on the wound healing process and tissue structure at the soft tissue-abutment interface were observed for all groups. The peri-implant soft tissue of TC4-H/Nano at the healing time points of two (Fig. 6a-c) and four weeks (Fig. 6d-f) are illustrated.

After two weeks of healing, the peri-implant mucosa was separated from the abutment surface by a gap (Fig. 6a). The proliferation of the epithelium and JE was observed in the soft tissue margin. A newly formed loose CT zone with fewer fibers was rich in inflammatory cells (Fig. 6c). The peri-implant mucosa maintained close contact with the abutment surface after four weeks of healing (Fig. 6d). A BE was formed, and the number of inflammatory cells decreased. The loose CT became well-organized and was replaced by large portions of collagen fibers. There were more fibroblasts and fewer inflammatory cells in the CT.

### Histometric measurements

Descriptive analyses of the peri-implant mucosa heights, including epithelial and CT dimensions assessed at two and four weeks of healing, are illustrated in Table 2. The SE length was approximately 0.6–0.7 mm in all the groups. The BW around the abutment was formed as early as two weeks, and the dimensions were near 3 mm in all groups. Except for SE, the other soft tissue dimensions at four weeks were slightly longer than those at two weeks without a statistically significant difference.

At four weeks of healing, the JE length in the standard abutment group (1.3 ± 0.3 mm) was longer than that in the TC4-H/Nano (0.9 ± 0.3 mm), while the CT length in the standard abutment (1.9 ± 0.3 mm) was shorter than that in the TC4-H/Nano (2.1 ± 0.6 mm). However, no statistically significant differences were observed between all groups.

**Table 2:** Descriptive analysis of histometric measurements (mean ± SD).

Material	SE (mm)	JE (mm)	CT (mm)	BE (mm)	BW (mm)	PMH (mm)
2 weeks						
TC4-M	0.61 ± 0.23	1.06 ± 0.20	1.82 ± 0.74	1.68 ± 0.26	2.88 ± 0.85	3.50 ± 1.00
TC4-Nano	0.68 ± 0.22	0.95 ± 0.42	1.74 ± 0.39	1.64 ± 0.54	2.70 ± 0.73	3.38 ± 0.88
TC4-H/Nano	0.61 ± 0.27	1.00 ± 0.26	1.93 ± 1.10	1.61 ± 0.30	2.93 ± 1.33	3.54 ± 1.21
4 weeks						
TC4-M	0.56 ± 0.24	1.29 ± 0.27	1.89 ± 0.28	1.86 ± 0.34	3.19 ± 0.46	3.75 ± 0.60
TC4-Nano	0.63 ± 0.29	0.98 ± 0.39	1.88 ± 1.04	1.61 ± 0.41	2.86 ± 1.07	3.49 ± 1.23
TC4-H/Nano	0.71 ± 0.49	0.94 ± 0.29	2.07 ± 0.55	1.65 ± 0.73	3.01 ± 0.72	3.72 ± 1.03

Abbreviations: SE, sulcular epithelium; JE, junctional epithelium; CT, connective tissue; BE, barrier epithelium; BW, biological width; PMH, peri-implant mucosa height.

### Collagen fiber orientation

**Fig. 7:**
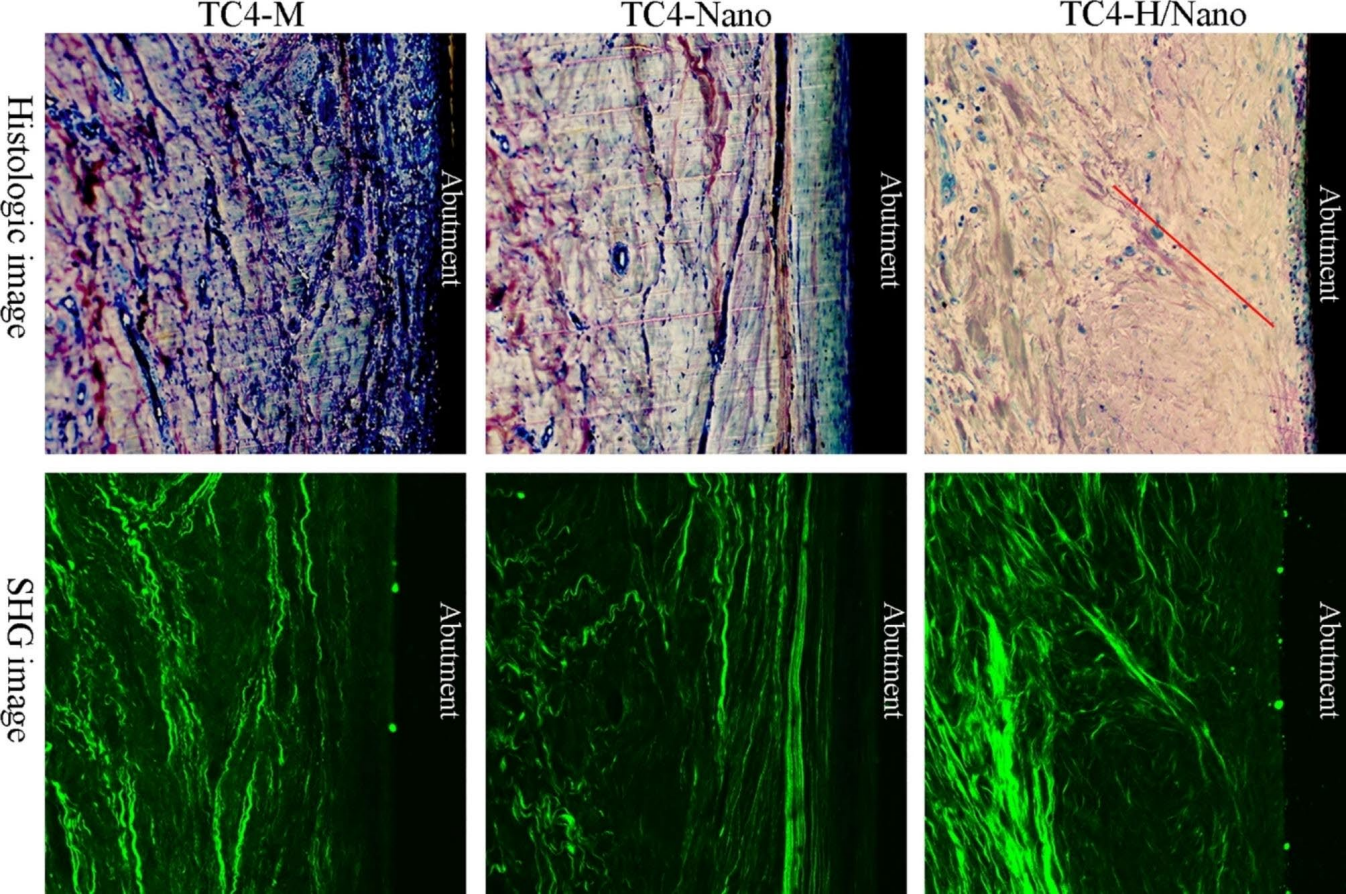
After four weeks of wound healing, histologic and SHG images of connective tissue (CT) adjacent to the abutment surface in all three groups. Red line: orientation of collagen fibers.

Images of peri-implant collagen structure and fiber orientation observed by optical and SHG microscopy are shown in Fig. 7. After four weeks of wound healing, loose tissue structure and less fiber composition were observed in the CT attached to TC4-M. However, adjacent to the TC4-Nano and TC4-H/Nano abutment surfaces, histological images showed a dense CT with more collagen fibers formed. SHG images showed that while collagen fibers in TC4-M and TC4-Nano groups ran parallel to the abutment surface, fibers adjacent to TC4-H/Nano tended to extend and were partially oriented perpendicular to the surface.

## Discussion

This study was designed to evaluate the possible advantages of a superhydrophilic nanotubular abutment surface over a machined surface regarding histometric soft tissue dimensions and collagen fiber orientation at the early stage of soft tissue integration.

Unlike our previous studies on pure titanium modification, the nanotubes on the TC4 alloy surface were nonuniform. Owing to the existence of the α and β phases in the TC4 alloy, an uneven etching rate in an electrolyte containing F was expected. The substrate of the β phase was enriched with vanadium oxides, resulting in a high solubility [37, 38]. Therefore, shorter nanotubes were formed in the β-phase (Fig. 4a, white arrows).

Our previous in vitro study demonstrated that the hydrogenated nanotubular surface can promote wound healing of gingival fibroblasts [21]. Costantini et al. [39] proved that the wound healing phase could be accelerated by driving the transition from the inflammation to the tissue repair. In the present in vivo study, we observed less inflammatory cells around abutments at 4 weeks. In order to better understand the wound healing around the nanotubular/superhydrophilic surface, a comprehensive analysis of the process of inflammation, proliferation and reconstruction in vivo should be conducted in the future.

In this in vivo experiment, the soft tissue dimensions surrounding the modified abutments were evaluated using histometric analysis. Our results revealed a relatively stable BW during the 2–4 weeks. These findings are consistent with those observed in another beagle study [26]. However, Berglundh et al. [27] and Tomasi et al. [40] suggested that it may take 6–8 weeks of healing to develop the BW in the transmucosal region. To date, definitive conclusions have not been attained.

Most studies have proved that surface modification (morphological, chemical, and biological modification) of materials at the transmucosal portion has limited influence on soft tissue dimensions [29, 32, 41]. In contrast, another study revealed that a longer CT seal developed in AO and AO + polydopamine groups (*p* < 0.05) [42]. It is worth noting that our present study showed a longer CT length surrounding the superhydrophilic nanotubes, combined with a shorter epithelium after four weeks of healing. However, this tendency was not statistically significant, which could be attributed to the small sample size. Schwarz et al. [43] designed a proof-of-concept clinical study for evaluating the performance of hydrophilic abutment surfaces in humans. Although not statistically significant, the study indicated quantitative and qualitative improvements in peri-implant soft tissue attachment to hydrophilic abutments. Therefore, we speculated that the superhydrophilic abutment surface could promote CT attachment, thus inhibiting the apical migration of the JE. However, further studies may be required based on a larger sample size and extended observation time.

SHG microscopy was innovatively employed for peri-implant collagen fiber observation in this study. Direct visualization of the tissue structure, orientation, and polarization of chiral proteins can be achieved by SHG with high resolution and specificity [44], which is derived from the interaction of near-infrared light with non-centrosymmetric tissues such as collagen and myosin. Although SHG is commonly applied to scar tissue and tumors [33, 45], it has not been used to image peri-implant tissues in oral. Our SHG results demonstrated that collagen fibers were partially perpendicular to the hydrogenated abutment, indicating that a superhydrophilic surface was conducive to the functional attachment of CT. Nevertheless, quantitative analysis of the orientation should be conducted in the future.

Schwarz et al. [26] speculated that hydrophilicity could be more important for collagen fibers orientation than surface topography. Similarly, a previous investigation indicated that the number of vertical fibers around a superhydrophilic implant increased compared to that around a machined abutment [32]. Overall, a superhydrophilic surface may be beneficial for the formation of a soft tissue barrier and subsequently reduce the risk of peri-implantitis. However, further in vivo experiments should be conducted to investigate the superhydrophilic surface regarding the prevention of peri-implant mucositis and peri-implantitis in a peri-implantitis model.

Although surface modification of transmucosal materials might have little influence on soft tissue dimensions, it could improve the healing process and promote peri-implant soft tissue health in long term [46, 47] by reducing inflammation [19], promoting vascularization and expression of adhesion-related biomolecules [48, 49], and collagen fiber attachment [50]. A recent review also indicated that some properties of transmucosal abutments might affect soft tissue attachment and stability [7].

In this study, limited information on cellular and matrix levels such as cell interactions, immunological factors, and connective tissue adhesion-related mechanisms was available only through histologic and histometric analyses. Therefore, to better understand the mechanisms of soft tissue integration, future researches should be conducted to comprehensively analyze the impact of superhydrophilic abutments surfaces on the clinical, histopathological, immunohistochemical, and molecular biological aspects of peri-implant soft tissue. In addition, further studies based on larger sample sizes, extended observation times, and peri-implantitis models may be required.

## Conclusions

Within the limitations of this study, it can be concluded that abutments with nanotubular and superhydrophilic surfaces compared with machined titanium alloy surfaces displayed a similar impact on healing conditions and peri-implant soft tissue dimensions but with a tendency to increase CT height and perpendicular collagen fibers. However, this tendency was not statistically significant. Our study implied that imparting nanotubular/superhydrophilic topography to conventional TC4 abutments is a promising modification to enhance soft tissue sealing.

## Data Availability

All data generated or analysed during this study are included in this published article.

## Abbreviations

TC4-M: machined Ti-6Al-4V alloy abutments
Nano: nanotubes
H/Nano: hydrogenated nanotubes
CT: connective tissue
MBL: marginal bone loss
ECM: extracellular matrix
FE-SEM: field-emission scanning electron microscopy
EDS: energy dispersive X-ray spectroscopy
XPS: X-ray photoelectron spectroscopy
AFM: atomic force microscopy
ARRIVE: animal research:reporting of *in vivo* experiments
TSV: tapered screw-vent
PM: peri-implant mucosa margin
cJE: most coronal point of the junctional epithelium
aJE: apical extension of the junctional epithelium
BIC: most coronal portion of the bone-implant contact
SE: sulcular epithelium
JE: junctional epithelium
BE: barrier epithelium
BW: biological width
PMH: peri-implant mucosa height
SHG: second harmonic generation
SD: standard deviation
ANOVA: one-way analysis of variance
AO: anodic oxidation
